# Tailored generation of insulin producing cells from canine mesenchymal stem cells derived from bone marrow and adipose tissue

**DOI:** 10.1038/s41598-021-91774-3

**Published:** 2021-06-11

**Authors:** Watchareewan Rodprasert, Sirirat Nantavisai, Koranis Pathanachai, Prasit Pavasant, Thanaphum Osathanon, Chenphop Sawangmake

**Affiliations:** 1 Inter-Disciplinary Program of Pharmacology, Graduate School, Chulalongkorn University, Bangkok, Thailand; 2 Veterinary Pharmacology and Stem Cell Research Laboratory, Veterinary Stem Cell and Bioengineering Innovation Center (VSCBIC), Faculty of Veterinary Science, Chulalongkorn University, Bangkok, Thailand; 3 Veterinary Stem Cell and Bioengineering Research Unit, Faculty of Veterinary Science, Chulalongkorn University, Bangkok, Thailand; 4 Department of Anatomy and Center of Excellence for Regenerative Dentistry (CERD), Faculty of Dentistry, Chulalongkorn University, Bangkok, Thailand; 5 Department of Pharmacology, Faculty of Veterinary Science, Chulalongkorn University, Bangkok, Thailand

**Keywords:** Gene expression analysis, Stem cells, Regeneration, Stem-cell differentiation, Transdifferentiation

## Abstract

The trend of regenerative therapy for diabetes in human and veterinary practices has conceptually been proven according to the Edmonton protocol and animal models. Establishing an alternative insulin-producing cell (IPC) resource for further clinical application is a challenging task. This study investigated IPC generation from two practical canine mesenchymal stem cells (cMSCs), canine bone marrow-derived MSCs (cBM-MSCs) and canine adipose-derived MSCs (cAD-MSCs). The results illustrated that cBM-MSCs and cAD-MSCs contain distinct pancreatic differentiation potential and require the tailor-made induction protocols. The effective generation of cBM-MSC-derived IPCs needs the integration of genetic and microenvironment manipulation using a hanging-drop culture of *PDX1*-transfected cBM-MSCs under a three-step pancreatic induction protocol. However, this protocol is resource- and time-consuming. Another study on cAD-MSC-derived IPC generation found that IPC colonies could be obtained by a low attachment culture under the three-step induction protocol. Further, Notch signaling inhibition during pancreatic endoderm/progenitor induction yielded IPC colonies through the trend of glucose-responsive C-peptide secretion. Thus, this study showed that IPCs could be obtained from cBM-MSCs and cAD-MSCs through different induction techniques. Also, further signaling manipulation studies should be conducted to maximize the protocol’s efficiency.

## Introduction

Diabetes is a major metabolic disease that affects not only people around the world but also human’s companion animals, mostly dogs and cats^1–3^. Pathophysiologically, it is classified into two main types, I and II, characterized by the absence or presence of intact beta-cells, respectively^2,4^. Type I diabetes refers to immune-mediated beta-cell destruction that causes endogenous insulin depletion, while type II involves insulin secretion defects and/or insulin resistance^1,4^. Although, diabetes treatment seems well-established, adverse events and compromised clinical efficiency have been reported periodically^3,5^. The trend of regenerative treatment has been introduced to address these issues, starting from cadaveric islet transplantation in diabetes type I patients, which is called the Edmonton protocol. It results in the long-term omission of exogenous insulin administration^6–8^. However, two main obstacles have been identified, donor shortages and immunosuppressants’ side effects, making stem cell (SC)-based regenerative approach be the potential clinical candidate^6–9^.

The concept of SC-derived insulin-producing cell (IPC) transplantation for treating diabetes has been conceptually approved in animal models*.* However, it comes with further challenges on finding potential candidate cell sources and establishing efficient IPC production platforms that are clinically applicable^10–12^. Although, the study of IPC production using human SCs has widely been studied and is well-established, knowledge of IPC generation aiming for veterinary application is still lacking. A few reports have suggested the induction of canine somatic cells and canine mesenchymal stem cells (cMSCs) toward IPCs in vitro^13,14^. These generated IPCs are formed as cell aggregates attached to the culture surface, which might cause some difficulties during cell harvesting and processing for transplantation. To earn the clinically applicable IPCs, a three-dimensional (3D) structure of IPCs floating or suspended in culture vessels would be required to ease the harvesting and encapsulating processes^15^. To address this issue, the integrative induction protocols aiming for the pancreatic differentiation of canine bone marrow-derived MSCs (cBM-MSCs) and canine adipose-derived MSCs (cAD-MSCs) were established in this study. Both cells have been previously isolated, characterized, and studied for their potential application in some diseases^16–20^. The protocols in this study aimed for the delivery of the 3D colony structure of the generated IPCs. Notch signaling manipulation was additionally conducted in the potential protocol for maximizing induction efficiency. The results will be the crucial platform supporting the IPC generation, which eventually benefits the establishment of clinical protocols for both veterinary and human applications.

## Results

### cBM-MSC and cAD-MSC characterization

**Figure 1:**
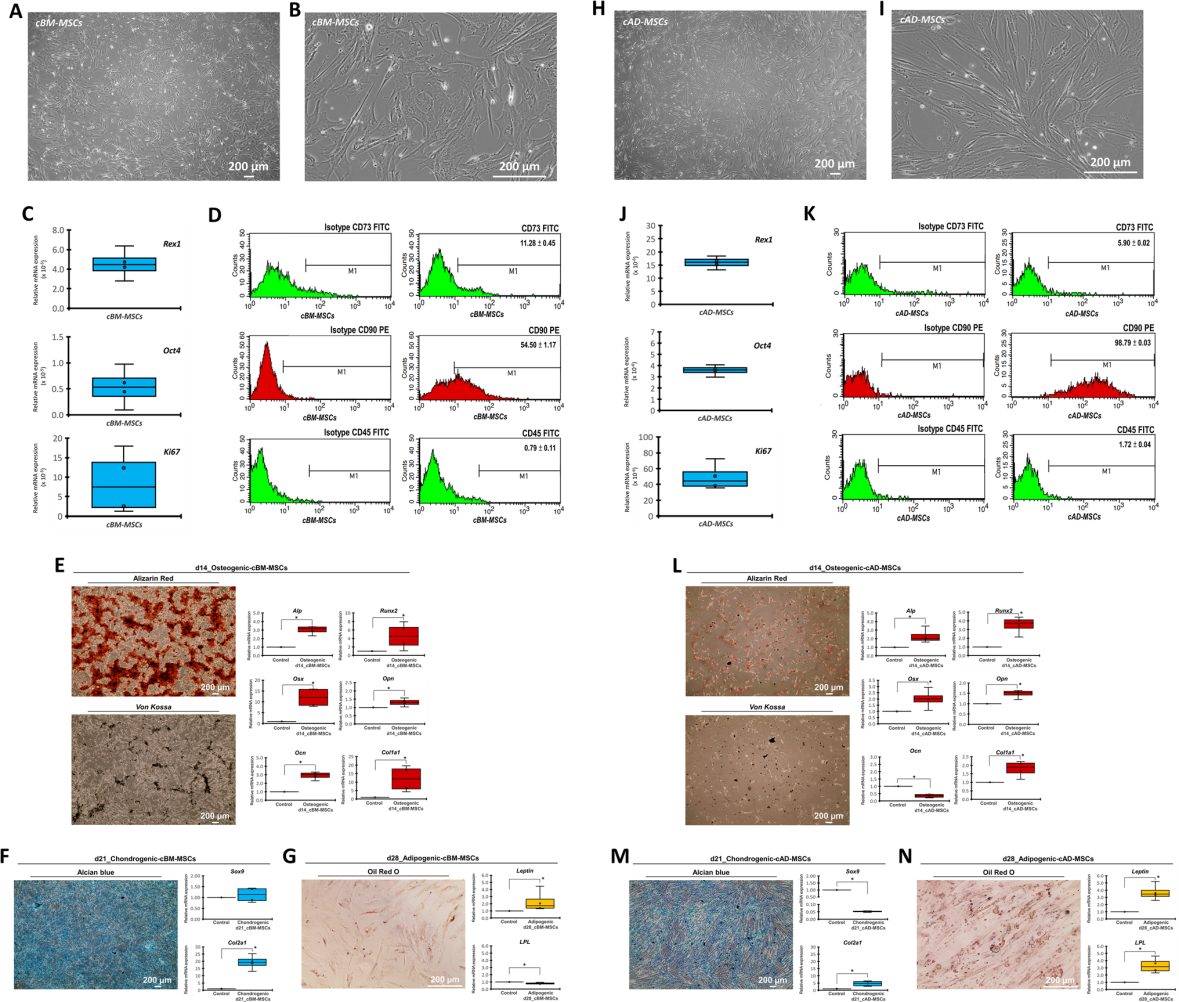
cBM-MSC and cAD-MSC characterization. Morphological appearances of cBM-MSCs (**A**,**B**) and cAD-MSCs (**H**,**I**) were observed under phase-contrast microscope with magnification of 40X and 200X. mRNA expressions regarding stemness and proliferation markers (**C**,**J**) were determined by RT-qPCR. mRNA expression was normalized with a reference gene. The MSC-related surface markers were analyzed using flow cytometry (**D**,**K**). Osteogenic differentiation potential at day 14 post-induction was determined by Alizarin Red S and *Von Kossa* staining, and the osteogenic mRNA marker expression was analyzed by RT-qPCR (**E**,**L**). Chondrogenic differentiation potential at day 21 post-induction was determined by Alcian blue staining, and chondrogenic mRNA markers were determined (**F**,**M**). Adipogenic differentiation potential at day 28 post-induction was determined by Oil Red O staining, and adipogenic mRNA markers were determined (**G**,**N**). mRNA expression was normalized with a reference gene and undifferentiated control. Bars indicate a significant difference (*, *p* value < 0.05).

The isolated cBM-MSCs (Fig. 1A,B) and cAD-MSCs (Fig. 1H,I) showed a fibroblast-like appearance on the 2D culture. The mRNA expressions of stemness-related markers (*Rex1* and *Oct4*) and the proliferation marker (*Ki67*) were detected (Fig. 1C,J). An MSC-related surface marker analysis by flow cytometry showed that both cells contained high proportion of Cd90^+^ cells, while the proportion of Cd73^+^ cells was relatively low. The expression of the hematopoietic surface marker (Cd45) was considered absent in both cells (Fig. 1D,K).

Both cells illustrated the in vitro osteogenic differentiation potential upon the 14-day induction protocol regarding extracellular matrix (ECM) mineralization as demonstrated by Alizarin Red S and *Von Kossa* staining and the osteogenic mRNA marker expression (*Alp*, *Runx2*, *Osx*, *Opn*, *Ocn,* and *Col1a1*) (Fig. 1E,L).

A differentiation potential toward chondrogenic and adipogenic lineages of both cell types was also shown as positive glycosaminoglycans staining by Alcian Blue dye and the expression of chondrogenic mRNA markers (*Sox 9* and *Col2a1*) (Fig. 1F,M), along with oil droplet staining by Oil Red O dye and the expression of adipogenic mRNA markers (*Leptin* and *LPL*) (Fig. 1G,N).

The results revealed the MSC-related characteristics of the isolated cBM-MSCs and cAD-MSCs.

### Generation of IPCs from cBM-MSCs requires a 3D culture condition

**Figure 2:**
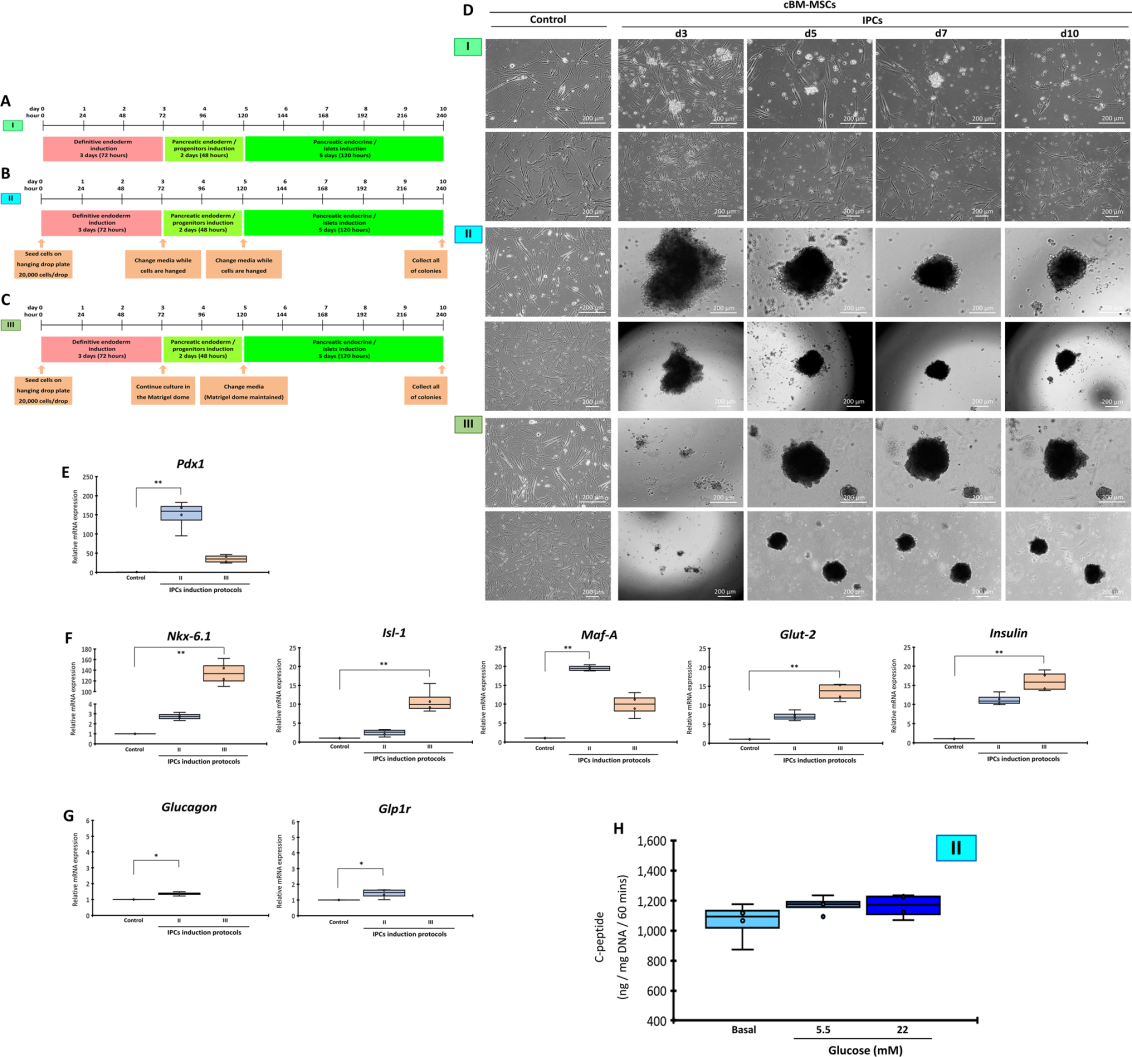
Generation of cBM-MSC-derived IPCs by microenvironment manipulation. The diagrams of three culture techniques used for the generation of cBM-MSC-derived IPCs are shown: (I) low attachment (**A**), (II) hanging-drop (**B**), and (III) hydrogel-embedded (**C**) culture techniques. Morphological appearances of cells that underwent each of induction technique were observed under a phase-contrast microscope with 100X and 200X magnification (**D**). mRNA markers relating to pancreatic endoderm (**E**), pancreatic beta-cell (**F**), and pancreatic-relating markers (**G**) were analyzed by RT-qPCR. mRNA expression was normalized with reference gene and undifferentiated control. Functional testing by glucose-stimulated C-peptide secretion (GSCS) was illustrated (H). Bars indicate a significant difference (*, *p* value < 0.05; **, *p* < 0.01).

To generate IPC colonies from cBM-MSCs, three different culture techniques were investigated (Fig. 2A–C). In all culture techniques, three pancreatic induction media were used as a microenvironment-manipulating or small-molecule-inducing approach. The results, as illustrated in Fig. 2D, showed that suspending the cells in a low attachment culture dish (Protocol I) was unable to deliver IPC colonies, while maintaining the cells using the hanging-drop technique (Protocol II) could successfully generate IPC colonies with 50–200 µm in diameter. However, the colonies seemed loose cell aggregates. Further investigation was performed by maintaining the colonies collected from the hanging-drop culture in the Matrigel-embedded culture condition (Protocol III). Although the generated colonies were dense and compact, they could not maintain the colony structure after gel digestion using the Cell Recovery Solution, making them unable to be harvested for further functional testing.

A comparison of the pancreatic mRNA markers of the generated IPC colonies revealed that colonies from Protocol II expressed a higher pancreatic endoderm marker (*Pdx1*) but lower pancreatic beta-cell markers (*Nkx-6.1*, *Isl-1*, *Glut-2*, and *Insulin*) compared with those from Protocol III (Fig. 2E,F). However, the mRNA expression of pancreatic-relating markers (*Glucagon* and *Glp1r*) was not detected in Protocol III (Fig. 2G).

Further functional testing showed that IPC colonies collected from Protocol II secreted C-peptide under a basal condition but could not produce a significant response upon low (5.5 mM) and high (22 mM) glucose stimulation. There was only a trend of increased C-peptide secretion compared to basal control (Fig. 2H).

Thus, generating IPCs from cBM-MSCs using the microenvironment-manipulating/small-molecule-inducing approach required a 3D culture condition. However, the generated IPCs showed limited functions and maturity.

### Overexpression of *PDX1* fails to generate IPCs from cBM-MSCs

**Figure 3:**
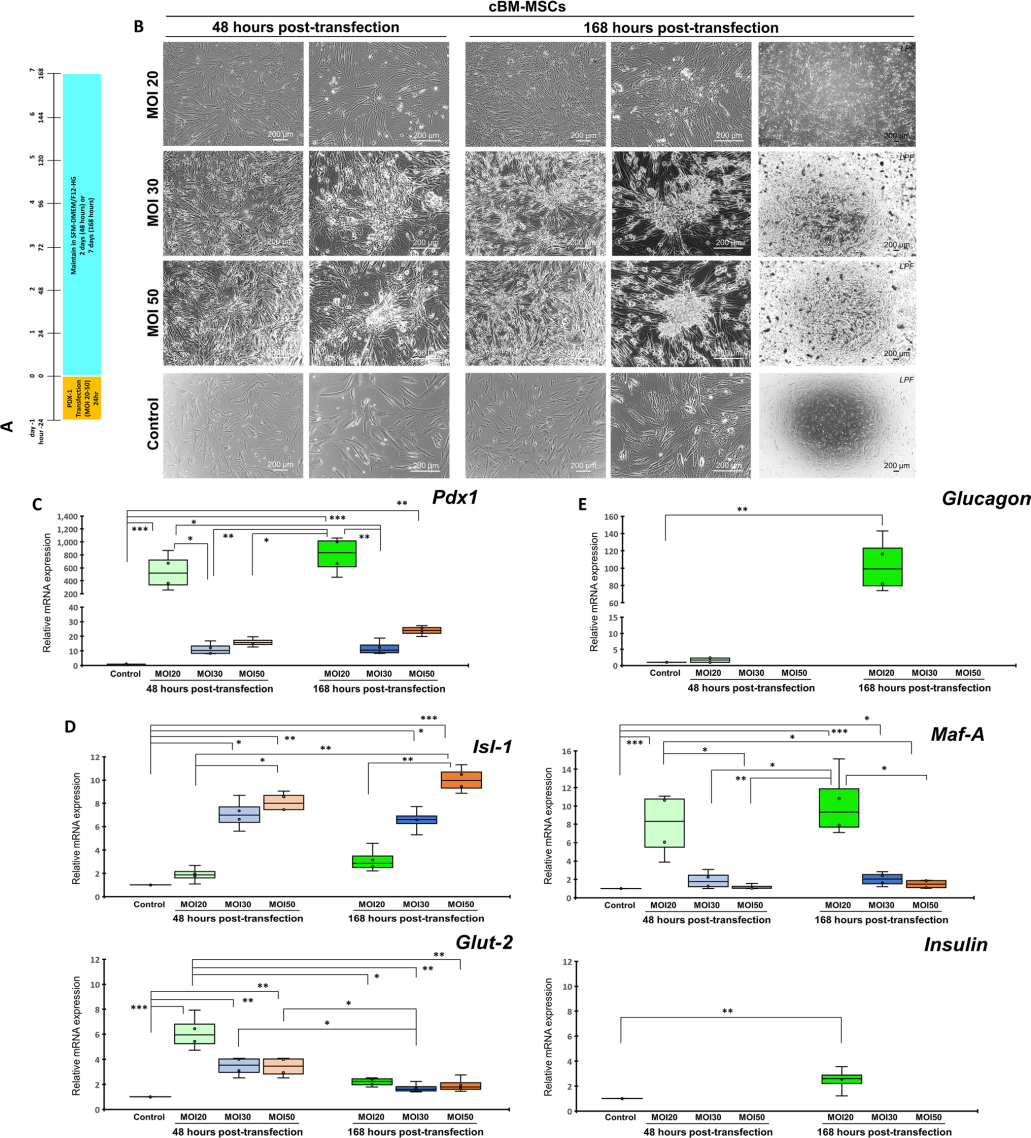
Generation of cBM-MSC-derived IPCs by genetic manipulation. Diagram of the *PDX1* transfection for the generation of cBM-MSC-derived IPCs is shown in (**A**). Morphological appearances of cells that underwent each of transfection condition were observed under a phase-contrast microscope with magnification of 40X, 100X, and 200X (**B**). mRNA markers relating to pancreatic endoderm (**C**), pancreatic beta-cell (**D**), and pancreatic-relating markers (**E**) were analyzed by RT-qPCR. mRNA expression was normalized with a reference gene and undifferentiated control. Bars indicate a significant difference (*, *p* value < 0.05; **, *p* < 0.01; ***, *p* < 0.001).

Further generating IPCs from cBM-MSCs using genetic manipulation was conducted through overexpression of the pancreatic commitment regulator, *PDX1*. The lentiviral vector carrying *PDX1* was transfected into cBM-MSCs at multiplicity of infection (MOI) 20, 30, and 50 (Fig. 3A). The results showed that all transfected cells started forming loose cell aggregates since 48-h post-transfection. Then, at 168-h post-transfection, transfected cells at MOI 20 formed small-size cell clusters (< 50 µm in diameter), while those transfected at MOI 30 and 50 formed medium- to large-size cell clusters (100–200 µm in diameter). None of them formed a floating colony-like structure (Fig. 3B).

A further analysis of pancreatic mRNA markers showed that transfected cells at MOI 20 significantly illustrated high expressions of the pancreatic endoderm marker (*Pdx1*) and some pancreatic beta-cell markers (*Maf-A*, *Glut-2*, and *Insulin*) compared with those transfected at MOI 30 and 50 (Fig. 3C,D). However, the alpha-cell hormonal marker (*Glucagon*) was significantly expressed in MOI 20 transfection (Fig. 3E), while *Glp1r* was not detected in all groups. The results suggested that *PDX1* overexpression could not successfully generate IPC colonies from cBM-MSCs in terms of pancreatic islet morphology and genotype.

### Integration of PDX1 overexpression with the 3D culture effectively generates IPCs from cBM-MSCs

**Figure 4:**
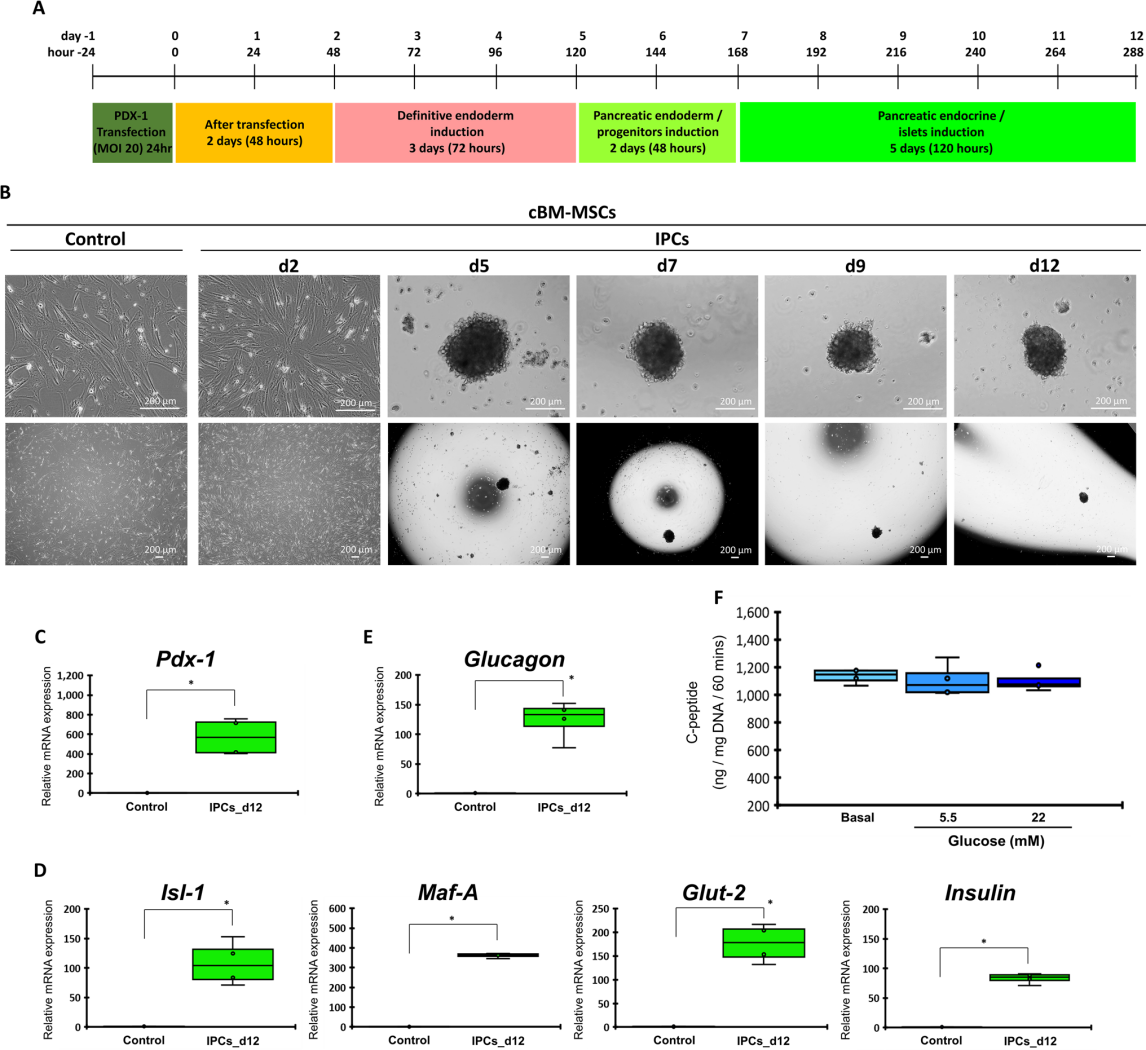
Generation of cBM-MSC-derived IPCs by integrating genetic and microenvironment manipulation. Diagram of culture technique used for the generation of cBM-MSC-derived IPCs is shown in (**A**). Morphological appearances of cells that underwent induction technique were observed under a phase-contrast microscope with magnification of 40X and 200X (**B**). mRNA markers relating to pancreatic endoderm (**C**), pancreatic beta-cell (**D**), and pancreatic-relating markers (**E**) were analyzed by RT-qPCR. mRNA expression was normalized with a reference gene and undifferentiated control. Functional testing by glucose-stimulated C-peptide secretion (GSCS) was illustrated (**F**). Bars indicate a significant difference (*, *p* value < 0.05).

To effectively generate IPCs from cBM-MSCs, a combination of genetic and microenvironment-manipulating approaches was used. Cells were transfected with the lentiviral vector carrying human *PDX1* at MOI 20 and then maintained with the three-step induction protocol under the 3D culture condition (hanging-drop technique) (Fig. 4A). The results illustrated that IPC colonies started forming since day 5 of the induction, and the size of colonies at day 12 was approximately 100–200 µm (Fig. 4B).

Pancreatic mRNA analysis showed that the pancreatic endoderm marker (*Pdx1*) and pancreatic beta-cell markers (*Isl-1, Maf-A*, *Glut-2*, and *Insulin*) were significantly upregulated (Fig. 4C,D). However, the alpha-cell hormonal marker (*Glucagon*) was highly expressed (Fig. 4E), while *Glp1r* was not detected. Functional testing also showed that IPC colonies secreted C-peptide under a basal condition, but they could not produce a dose-dependent response upon low (5.5 mM) and high (22 mM) glucose stimulation (Fig. 4F).

Thus, a combination of genetic and microenvironment-manipulating approaches effectively generated IPCs from cBM-MSCs with high pancreatic mRNA marker expressions, along with the ideal islet morphology. However, their functional property was still limited.

### Low attachment culture is efficient to generate IPCs from cAD-MSCs

**Figure 5:**
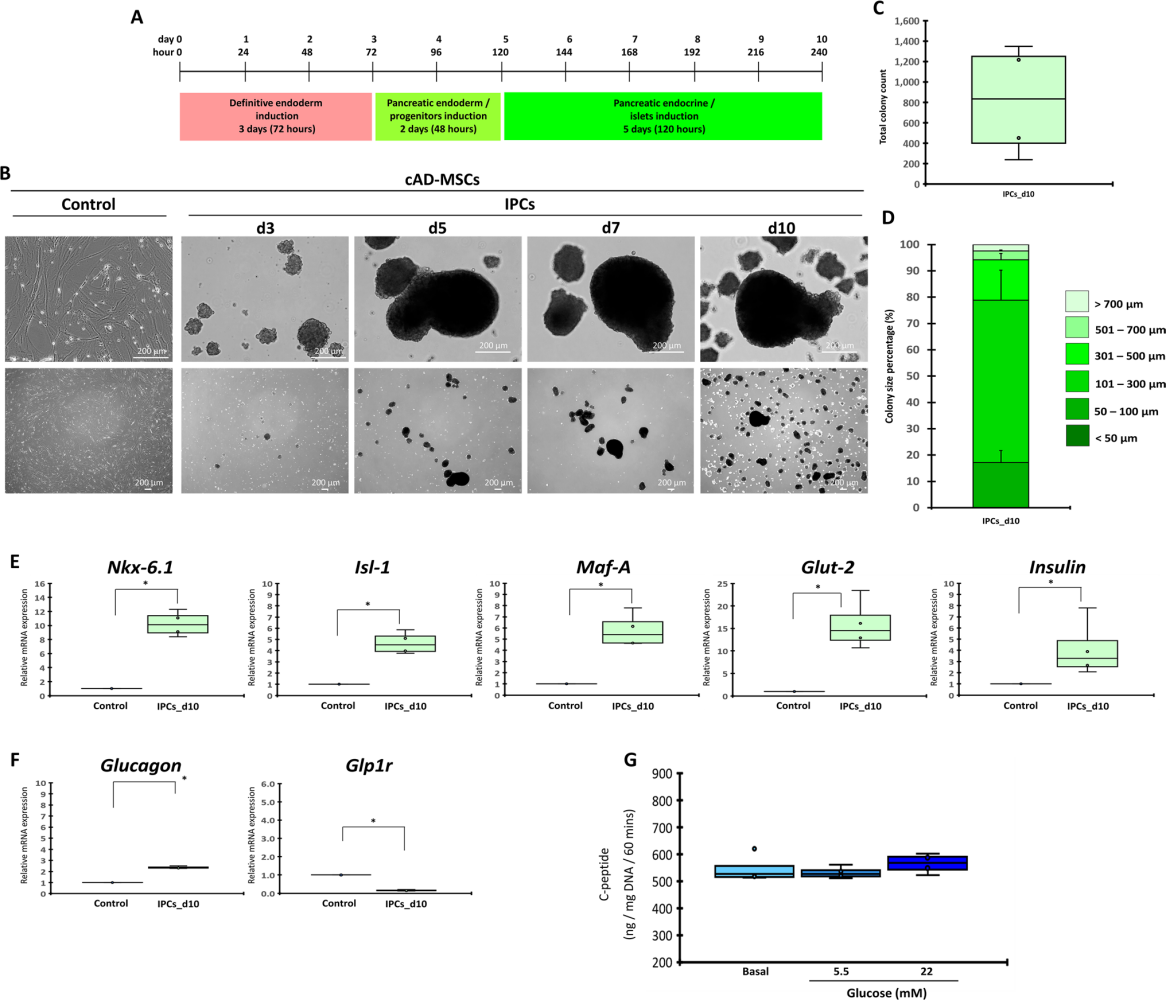
Generation of cAD-MSC-derived IPCs by microenvironment manipulation. Diagram of culture technique used for the generation of cAD-MSC-derived IPCs is shown in (**A**). Morphological appearances of cells that underwent induction technique were observed under phase-contrast microscope with magnification of 40X and 200X (**B**). Total colony number (**C**) and colony size proportion (**D**) were evaluated. mRNA markers relating to pancreatic beta-cell (**E**), and pancreatic-relating markers (**F**) were analyzed by RT-qPCR. mRNA expression was normalized with reference gene and undifferentiated control. Functional testing by glucose-stimulated C-peptide secretion (GSCS) was illustrated (**G**). Bars indicate a significant difference (*, *p* value < 0.05).

To generate IPCs from cAD-MSCs, the microenvironment-manipulating approach was used by suspending the cells onto low attachment culture dishes and maintaining the three-step induction media (Fig. 5A). It was quite interesting that cells formed colony-like structures since day 3 of the induction, and the colonies became denser and bigger during the culture period (Fig. 5B). At day 10, approximately 834 colonies (median) were obtained from 1 × 10^6^ seeding cells (Fig. 5C), and the colony size varied from < 50 µm to > 700 µm (Fig. 5D).

The analysis of the pancreatic mRNA expression revealed that pancreatic beta-cell markers (*Nkx-6.1, Isl-1, Maf-A*, *Glut-2*, and *Insulin*) were significantly upregulated (Fig. 5E). The alpha-cell hormonal marker (*Glucagon*) was a bit expressed, while *Glp1r* was downregulated (Fig. 5F). Functional testing showed that IPC colonies secreted C-peptide under a basal condition and showed a trend of glucose-responsive C-peptide secretion upon high (22 mM) glucose stimulation. However, it was not statistically significant compared to basal secretion (Fig. 5G).

The results suggested that the microenvironment-manipulating approach using the low attachment culture was efficient to generate IPCs from cAD-MSCs in terms of pancreatic islet characteristics. However, their functional property was still limited.

### Notch signaling optimization generates potential cAD-MSC-derived IPCs

The IPC induction protocol efficiency results have suggested that the generation of cAD-MSC-derived IPCs using the microenvironment-manipulating approach seemed to be the most efficient protocol in terms of 1) morphological appearance and colony number, 2) pancreatic mRNA marker expression, and 3) functional property. In this regard, Notch signaling optimization was performed to generate the potential cAD-MSC-derived IPCs using the protocol mentioned in our previous report^21^.

**Figure 6:**
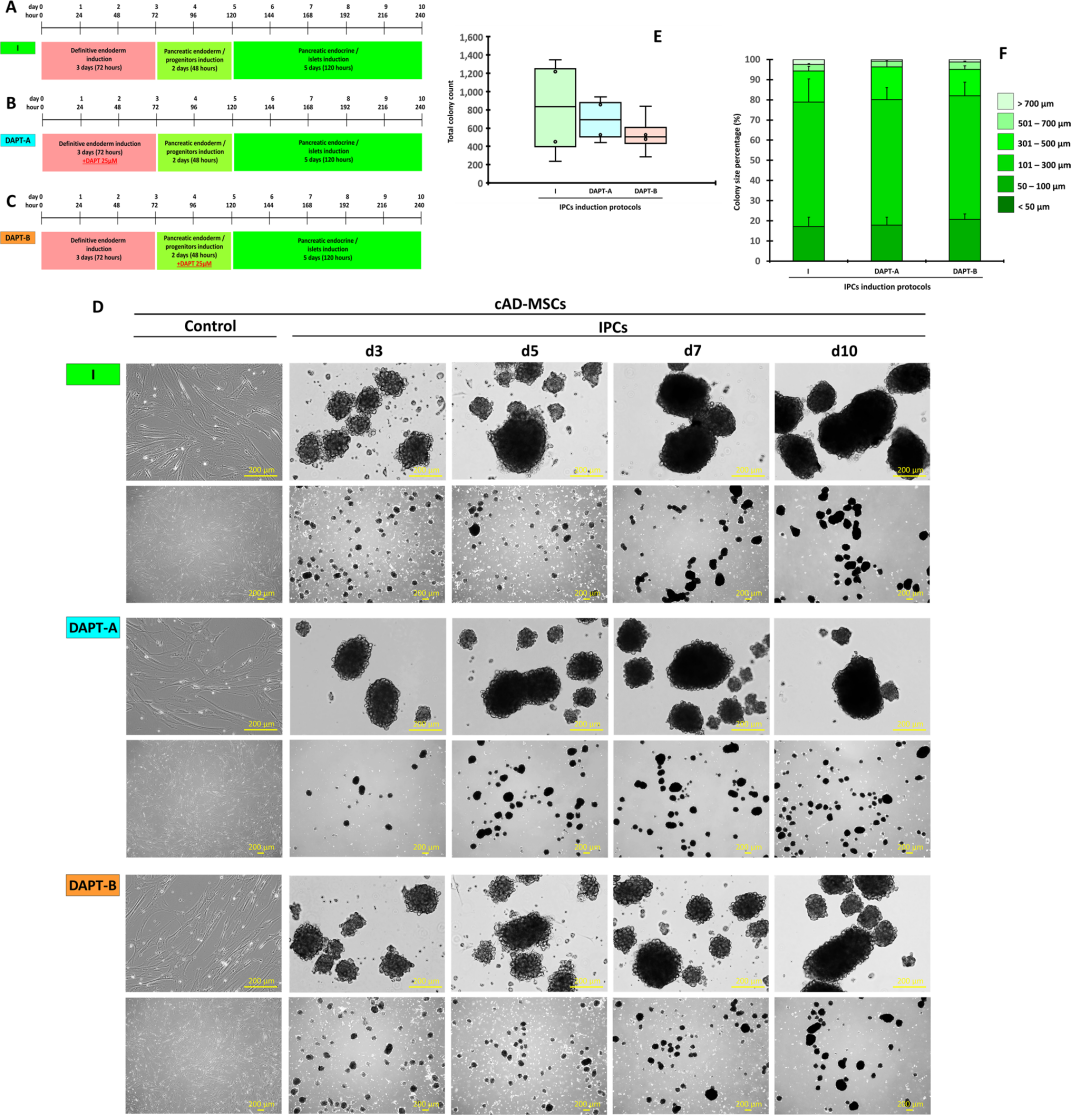
Generation of cAD-MSC-derived IPCs with Notch signaling manipulation. Diagrams of Notch signaling manipulation used for the generation of cAD-MSC-derived IPCs are shown (**A**–**C**). Morphological appearances of cells that underwent each of induction technique were observed under a phase-contrast microscope with magnification of 40X and 200X (**D**). Total colony number (**E**) and colony size proportion (**F**) were evaluated.

cAD-MSC-derived IPCs were generated using the optimized three-step induction protocol (Fig. 6A) with Notch signaling manipulation using a gamma-secretase inhibitor, DAPT, during definitive endoderm induction (DAPT-A) (Fig. 6B) or pancreatic endoderm/progenitor induction (DAPT-B) (Fig. 6C). The results showed that, in all conditions, cells started colony formation since day 3 post-induction. Then, the colony size and number increased during the induction period (Fig. 6D). The total colony counts (median) were 834, 691.5, and 504 colonies per batch (1 × 10^6^ seeding cells) for control, DAPT-A, and DAPT-B, respectively (Fig. 6E). It seemed that DAPT-B delivered more small-size colonies (< 50 µm and 50–100 µm), but the statistical difference was not recognized due to variation among groups (Fig. 6F).

**Figure 7:**
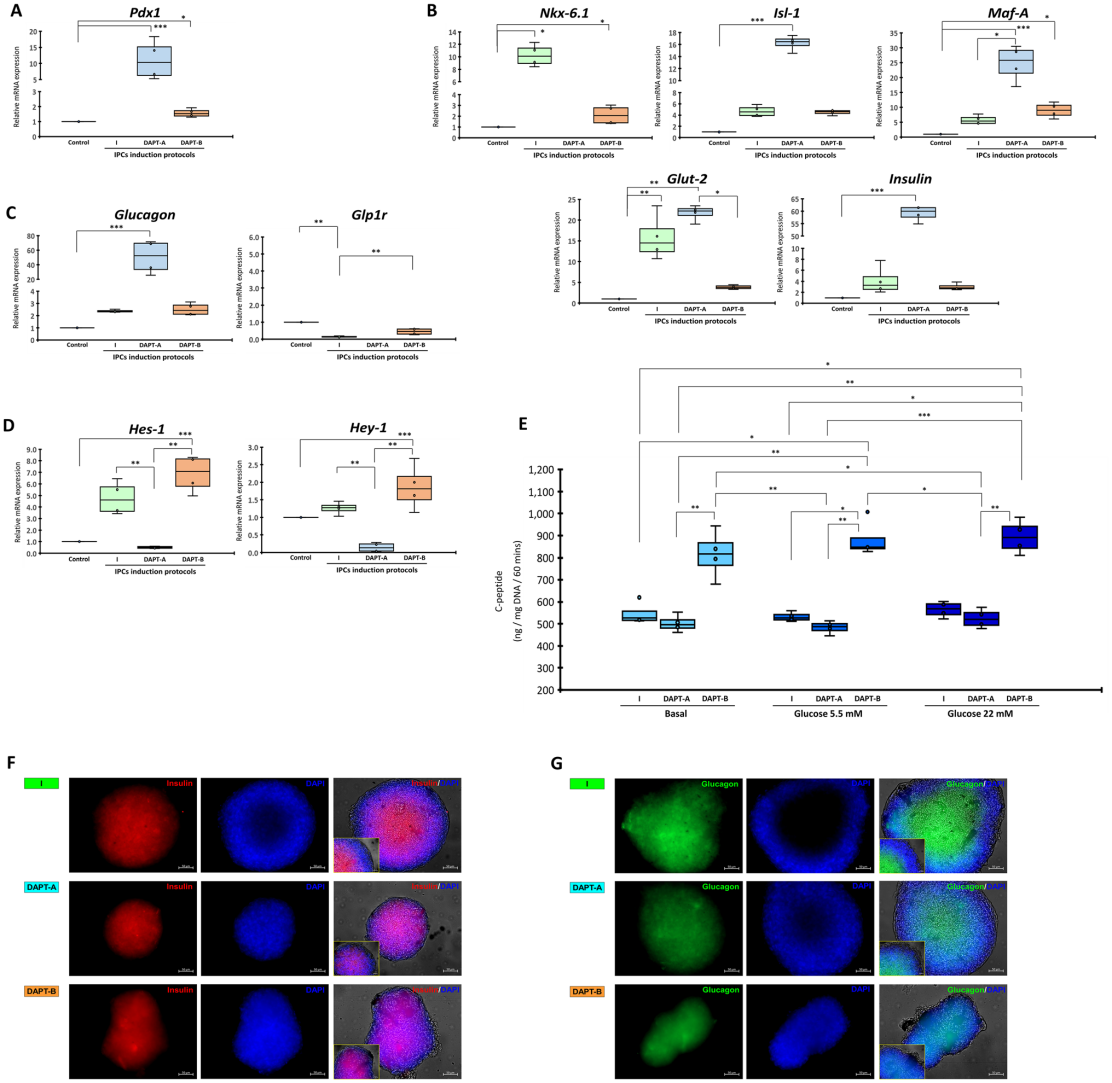
Generation of cAD-MSC-derived IPCs with Notch signaling manipulation. mRNA markers relating to pancreatic endoderm (**A**), pancreatic beta-cell (**B**), pancreatic-relating markers (**C**), and Notch target genes (**D**) were analyzed by RT-qPCR. mRNA expression was normalized with a reference gene and undifferentiated control. Functional testing by glucose-stimulated C-peptide secretion (GSCS) is illustrated in (**E**). Insulin (**F**) and Glucagon (**G**) protein expression were evaluated by immunocytochemistry staining. The results were observed under fluorescent microscope ZEISS Apotome.2 (Carl Zeiss, Germany) incorporated with Axio Observer Z1 and ZEN pro software (ZEISS International, Germany). Bars indicate a significant difference (*, *p* value < 0.05; **, *p* < 0.01; ***, *p* < 0.001).

The pancreatic mRNA analysis illustrated that cAD-MSC-derived IPCs from the DAPT-B condition significantly showed less pancreatic endoderm (*Pdx1*) and pancreatic beta-cell markers (*Isl-1, Maf-A*, *Glut-2*, and *Insulin*) than those from the DAPT-A condition (Fig. 7A,B). Additionally, the alpha-cell hormonal marker (*Glucagon*) of the DAPT-B group was much lower than that of the DAPT-A group. *Glp1r* was downregulated in all conditions (Fig. 7C). Interestingly, the analysis of Notch target genes *Hes-1* and *Hey-1* showed that the DAPT-B group showed significant upregulation of both genes compared with others (Fig. 7D). Functional testing showed that cAD-MSC-derived IPCs from the DAPT-B condition yielded the highest basal C-peptide secretion as well as the higher glucose-responsive C-peptide secretion upon low (5.5 mM) and high (22 mM) glucose stimulation compared with the control and DAPT-A groups. Due to variation between the groups, the statistical difference within each group was not found (Fig. 7E). An additional analysis on the protein expression of the crucial pancreatic endocrine hormones Insulin and Glucagon was performed using immunocytochemistry staining. The results suggested the expression of both proteins by the colonies collected from cAD-MSC induction (Figs. 7F,G). To affirm that the IPCs derived from cAD-MSC induction were able to secrete insulin upon culture medium maintenance, DMEMs supplemented with various glucose concentration (0, 5.56, and 25 mM) were used. The results suggested a similar trend of insulin secretion, as previously illustrated in the functional analysis (Supplementary Figure S1).

Taken together, the results suggested that cAD-MSC-derived IPCs could be efficiently generated using the microenvironment-manipulating approach with Notch optimization. The obtained IPCs from Notch inhibition during pancreatic endoderm/progenitor induction showed pancreatic islet/beta-cell characteristics and a positive trend of functional property.

## Discussion

As the proof-of-concept evidence for treating diabetes by regenerative therapy has been reported in human and animal models^9,22–25^, MSCs have been proposed as one of the promising resources for generating clinically applicable IPCs^26–29^. In this study, the pancreatic differentiation potential of cBM-MSCs and cAD-MSCs was evaluated to determine the feasibility of IPC formation in vitro and the potential of their clinical application. The cBM-MSCs and cAD-MSCs were isolated, cultured, and expanded using previous published protocols^17,18,30,31^. Their characteristics were similar to those described in previous reports, including fibroblast-like structures, mRNA expressions related to stemness and proliferation, and MSC-related surface marker expression, along with the multilineage differentiation potential toward osteogenic, chondrogenic, and adipogenic lineages^16–18,31–34^. In this study, we employed the flow cytochemistry analysis on the expression of representative MSC-related and hematopoietic surface markers (Cd73, Cd90, and Cd45) according to our recent publication^35^. The expression of Cd73 in both MSCs was relatively low, as mentioned in a previous report^36^. This evidence supported the consistency of the cMSCs’ properties used in this report.

In terms of IPC formation in vitro, various protocols employing either microenvironment or genetic manipulation have been reported^11,37–41^. The strategies used in these studies usually relied on the origin and pluripotency/multipotency of the cells^42–46^. Pluripotent SCs, ESCs, and iPSCs have high capability of pancreatogenesis in vitro^47–52^. However, due to their ethical and safety concerns, MSCs have been proposed as an alternative source of IPC generation^10,11,22,38,51,53–55^. According to the analysis of the differentiated cells derived from the induction protocol, a set of characterization protocol was designed based on the characteristics of the IPCs, including colony morphology and size, pancreatic mRNA marker expression, mature pancreatic protein marker immunocytochemistry staining, and functional analysis based on glucose-stimulated C-peptide secretion (GSCS) analysis. Due to the IPC colony structure, enzymatic digestion to get a single cell population for flow cytometry analysis may cause the damage to the colony structure and particular cell components^21,40^.

Here, we illustrated that cBM-MSCs and cAD-MSCs could be differentiated toward pancreatic lineage in vitro*.* However, each cell type had different pancreatic differentiation potential and required a tailor-made induction technique. For IPC generation by cBM-MSCs, it has been shown that the microenvironment-manipulating approach with a low attachment culture (2D culture) could not produce an islet-like cell aggregate in vitro, but it required a 3D culture technique for generating and maintaining the colony-like structure of IPCs. By using the hanging-drop culture technique, cBM-MSCs formed cell aggregates since day 3 post-induction, and then the size of the colony was increased, along with the expression of pancreatic mRNA markers. Further experiments showed that the Matrigel-embedded culture of the colonies derived from the hanging-drop culture could give a dense colony structure and higher levels of the pancreatic marker expression.

Previous publications reported that small molecule induction could imitate the environment during pancreatic endocrine development^10,21,48,56–61^. Generally, an in vitro pancreatic differentiation from SCs could be categorized into six differentiation stages: pluripotent/multipotent SCs, mesendoderm, definitive endoderm, pancreatic endoderm, pancreatic endocrine, and pancreatic beta-cells/IPCs^15,62^. In this study, activin A was used to mimic the effects of endogenous noggin for shortcutting the definitive endoderm-establishing step, as described in previous reports^37,62–67^. It was quite interesting that maintaining cBM-MSCs with pancreatic induction media in the low attachment culture was unable to form a colony-like structure, which is the natural pancreatic islet topology and crucial for an in vitro pancreatic differentiation^21,37,64,68–70^. Therefore, the 3D culture condition using hanging-drop and Matrigel-embedded culture techniques was used for generating the cBM-MSC-derived IPC colony. It was shown that the hanging-drop culture was an efficient technique for embryoid body/cell colony formation in vitro^71–74^, along with the natural/synthetic hydrogel-embedded culture that was one of the effective culture techniques used for organoid formation and expansion^75–79^. However, the issue on the cell number and the size of the colony obtained from the technique is critical. Further studies on the high capacity, high density hanging-drop culture may reflect the possibility of adopting this technique for real clinical application, as the evidence of necrotic core formation has been reported in large-size colony structures^80,81^. In this study, we demonstrated the successful IPC colony formation by these two culture techniques. However, it was quite difficult to collect and expand the IPC colonies since colony maintenance and medium changes for the hanging-drop culture were time-consuming. In addition, treating the Matrigel-embedded colonies with a hydrogel digestion solution (Cell Recovery Solution) caused colony dissociation. Further functional assay could only be performed for IPC colonies derived from the hanging-drop culture and found that the obtained IPC colonies could basally secrete C-peptide but not a significant response to glucose stimulation. In this study, secreted C-peptide was used as a representative marker for detecting secreted insulin from the IPCs due to the component of exogenous insulin added in a conditioned medium. Additional genetic manipulation was performed and showed that the overexpression of *PDX1* at MOI 20 could enhance pancreatic beta-cell marker expression but was unable to produce 3D IPC colonies.

These findings led to the integration of genetic and microenvironment manipulation using the hanging-drop culture of *PDX1*-transfected cBM-MSCs under three-step induction cocktails. The results demonstrated the effective formation of 3D IPC colonies with significant pancreatic marker expressions along with basal C-peptide secretion. Our findings were correlated to previous reports, showing that *PDX1* was an essential gene in the first hierarchy of pancreatic organogenesis progressing toward beta-cell maturation^62,82^. *PDX1-*positive cells were considered as the pancreatic progenitors for three pancreatic lineages, comprising endocrine, exocrine, and ductal cells^10^. It has been shown that the overexpression of *PDX1* by lentiviral vector into mouse MSCs could enhance IPC generation by triggering the morphological change from adherent spindle fibroblast-like cells toward the ball-like cell colonies^38,83^. For cBM-MSCs, we found that a 3D culture condition was required to form the IPC colony, which was considered as the native pancreatic islet morphology^40,68,70,84^. Thus, cBM-MSC-derived IPCs were able to obtain from the integrating protocol of genetic and microenvironment manipulation. However, the hanging-drop 3D culture technique was time- and labor-consuming, making it less clinically applicable.

Alternatively, cAD-MSCs have been proposed as the potential MSC candidate for regenerative diabetes therapy, as mentioned in previous reports^23,56,83,85–87^. We showed in this study that cAD-MSC-derived IPC colonies could efficiently be generated from a low attachment culture with the expression of crucial pancreatic mRNA and protein markers. A functional assay showed a basal C-peptide release with a trend of glucose-responsive C-peptide secretion in high glucose (22 mM) stimulation. Our finding was correlated with previous studies on the generation of pancreatic progenitors (PPs) and IPCs by AD-MSCs derived from human and animal resources^37,56,57,86,88^. Most of the IPC induction protocols used for AD-MSC induction relied on the concept of microenvironment induction, which reflects the trans-lineage differentiation potential of the cells^37,56,86,87^. In 2006, Timper et al. had initially proved the prospect of human AD-MSCs (hAD-MSCs) toward IPCs using single-step microenvironment manipulation^87^. After that, Chandra et al. published a three-step microenvironment manipulation protocol to induce murine AD-MSCs (mAD-MSCs) toward islet-like cell aggregates (ICAs)^37^. mAD-MSCs could be successfully committed to each stage of the pancreatic endocrine development regarding definitive endoderm, pancreatic endoderm, and pancreatic endocrine precursor, as illustrated by the upregulating pancreatic markers in each stage. These findings supported the pancreatic differentiation potential of AD-MSCs derived from various species.

It has been suggested that the promising regenerative therapy for diabetes relies on the availability and potential of stem cells used for generating PPs or IPCs, the efficiency of the induction protocol, and the potential application of an established transplantation platform. One of the potential transplantation platforms is cell or colony encapsulation, which requires the 3D colony structure of the IPCs that can be harvested after an in vitro production. This encapsulation platform can support and immobilize IPC colonies with the immunoisolating property against host immunity^15^. By comparing the potential clinical application, it seemed that cBM-MSC-derived IPCs showed less potential due to the complicated and time- or labor-consuming induction protocol. Therefore, cAD-MSC-derived IPCs were further optimized.

Various factors and signaling have been studied for their potential effects on IPC generation in vitro. In this regard, Notch signaling was of interest due to its significant effect during pancreatogenesis both in vivo and in vitro^89–92^. cAD-MSC-derived IPCs were generated using the optimized three-step induction protocol with Notch signaling manipulation by a gamma-secretase inhibitor, DAPT, during definitive endoderm or pancreatic endoderm/progenitor induction. We found that Notch inhibition during pancreatic endoderm/progenitor induction benefited the cAD-MSC-derived IPC production in terms of high basal C-peptide secretion and a positive trend of glucose-responsive C-peptide secretion. Our evidence also supported the ability of cAD-MSC-derived IPCs on glucose sensing and C-peptide secreting upon maintenance under the culture medium and the physiological buffer solution. These findings were also correlated with previous studies that Notch signaling played a biphasic role in pancreatogenesis during embryonic development. Downregulation of Notch is required for pancreatic endoderm commitment and *Pdx1*-postive pancreatic precursor expansion, while Notch upregulation is crucial for late-state pancreatic maturation^91–93^. Our previous study also showed that Notch inhibition during pancreatic endoderm induction by human dental pulp stem cells (hDPSCs) resulted in a large IPC colony production with high expression of *PDX1*, whereas the inhibition during the maturation stage caused the impairment of glucose-responsive C-peptide secretion^21^.

During pancreatogenesis, endocrine precursors formed clusters, which allowed cell-to-cell contact and the interaction, so called lateral inhibition. This led to the activation of Notch signaling and the regulation of the endocrine fate descended from *Pdx1*-positive progenitors^94–96^. Previous studies have confirmed the involvement of Notch signaling during endocrine progenitor fate commitment toward one of the pancreatic endocrine subtypes (beta- or alpha-cells)^96–98^. Notch inhibition by *HES1* shRNA could induce the redifferentiation of expanded human beta-cell-derived cells following the significant expansion of beta-cells in vitro and the upregulation of beta-cell-related genes^99^. However, Notch overactivation could limit the differentiation capability of fully matured IPCs by inhibiting the expression of the “pre-differentiation” gene by pancreatic progenitors^100^. These evidences also supported our findings that the cAD-MSC-derived IPCs could be generated in vitro, and the selective Notch signaling manipulation played the beneficial roles in colony production, pancreatic marker expression, and functional property.

According to our pilot study and previous publications, it was quite interesting that MSCs derived from different resources showed various differentiation potential. This might be due to the distinct cellular characteristics, behaviors, and underlying mechanisms regulating cell differentiation potential.

Our reports on the generation of IPCs from two sources of human dental tissue-derived MSCs, hDPSCs and human periodontal ligament stem cells (hPDLSCs), also showed the superior pancreatic differentiation potential of hDPSCs beyond hPDLSCs^21,40^. Recent publication from our team regarding bone tissue engineering also showed the distinct osteogenic differentiation potential by cBM-MSCs and canine dental pulp stem cells (cDPSCs) in vitro. To dissect the underlying mechanisms, proteomics-based systems biology analysis was applied, and it suggested that both cells required different signaling pathways and underlying mechanisms for regulating their osteogenic paths^35^.

In this study, we hypothesized that cBM-MSCs and cAD-MSCs behaved differently regarding the differentiation potential toward pancreatic lineages, so the sets of induction protocol have been designed for a particular cell type. Further mechanism and bioinformatics studies are indeed required for dissecting the underlying mechanisms governing pancreatic differentiation potential by both cells.

## Conclusion

In veterinary practice, a trend of SC-based regenerative treatment is not well established, and the potential therapeutic regimens have not been successfully validated. Regarding the SC-based diabetes treatment, part of the crucial steps is the establishment and validation of the potential cell resource and the induction protocol^101,102^.

In this study, we selected two potential MSC candidates for comparing the differentiation potential on the in vitro IPC generation and the potential application for further clinical trial. Both cells contained different properties in terms of cell characteristics, manipulation protocols, and IPC differentiation potential, reflecting a distinct potential application for future clinical trial and analysis. Our results suggested that cAD-MSCs might be a good MSC candidate for future application and the establishment of SC-based diabetes treatment for veterinary practice. Further studies focusing on maturation and the transplantation platform will fulfill the production of clinically applicable cMSC-derived IPCs.

## Materials and methods

### Cell isolation, culture, and expansion

All protocols were conducted in compliance with the ARRIVE guidelines and in accordance with the guidelines and regulations approved by the Institutional Animal Care and Use Committee (IACUC), the Faculty of Veterinary Science, and the Chulalongkorn University (Animal Use Protocol No.1531072). Four healthy dogs were used for each adipose or bone marrow sampling. According to the inclusion criteria, healthy dogs aged 5-10 years and weighted over 5 kg were recruited. Informed consent was taken from pets’ owners for the inclusion of the dogs in the study. cBM-MSCs were isolated from heparin-containing bone marrow aspirate following our previously published protocol^18^. Briefly, the cells were washed with Hank’s Balanced Salt solution (HBSS) (Thermo Fisher Scientific Corporation, USA), and then resuspended with high glucose Dulbecco’s Modified Eagle Medium: Nutrient Mixture F-12 (DMEM/F-12) (Thermo Fisher Scientific Corporation) supplemented with 10% fetal bovine serum (FBS) (Thermo Fisher Scientific Corporation), 1% GlutaMAX (Thermo Fisher Scientific Corporation), and 1% Antibiotics-Antimycotic (Thermo Fisher Scientific Corporation).

cAD-MSCs were isolated from biopsied adipose tissues. Tissues were minced and incubated with the Cell Recovery Solution (Corning, USA) for 2 h at 37℃ and then passed through a 70 µm strainer and washed twice with phosphate-buffered saline (PBS). Pellets were resuspended and seeded onto culture containers. Cells were maintained in high glucose DMEM (Thermo Fisher Scientific Corporation), supplemented with 10% FBS, 1% GlutaMAX, and 1% Antibiotics-Antimycotic.

Both cell types were maintained at 37℃ in humidified atmosphere with 5% CO_2_ and fresh air. Culture media was replaced every 48 h. Cells were subcultured when 80% confluence reached. Cells in passages 2–6 were used for the experiments.

### Characterization of cBM-MSCs and cAD-MSCs

The isolated cells were characterized by assessing mRNA expression regarding the stemness markers (*Rex1* and *Oct4*) and the proliferative marker (*Ki67*) by reverse transcription-quantitative polymerase chain reaction (RT-qPCR). MSC-related and hematopoietic surface markers were analyzed by flow cytometry. Cells were stained with mouse anti-Cd73 monoclonal antibody (Invitrogen, USA) and FITC-conjugated goat anti-mouse immunoglobulin (Ig) G secondary antibody (Bio-Rad, USA), PE-conjugated rat anti-Cd90 monoclonal antibody (eBioscience, USA), and FITC-conjugated mouse anti-Cd45 monoclonal antibody (Bio Legend, USA). Mouse IgG Isotype (Bio Legend), PE-conjugated rat IgG Isotype (Bio Legend), and FITC-conjugated mouse IgG Isotype (Bio Legend) were used as isotype control. A FACSCallibur flow cytometer with CellQuest software (BD Bioscience) was used for analysis.

Multilineage differentiation potential was assessed regarding osteogenicity, chondrogenicity, and adipogenicity. For osteogenic differentiation, the previously published induction protocol was used^17,21,103^. Briefly, the cells were seeded onto a 24-well culture plate (Corning, USA) in a concentration of 2.5 × 10^5^ cells/well. After 24 h, the cells were maintained in an osteogenic induction medium for 14 days. The osteogenic induction medium was a growth medium supplemented with 50 mg/mL L-ascorbic acid, 100 mM dexamethasone, and 10 mM β-glycerophosphate^21^. Osteogenic differentiation potential was analyzed according to extracellular matrix (ECM) mineralization by Alizarin Red S^104^ and *Von Kossa* staining^105^, and osteogenic-related mRNA marker expressions (*Alp*, *Runx2*, *Osx*, *Opn*, *Ocn*, and *Col1a1*) by RT-qPCR. Undifferentiated cells were used as controls.

For chondrogenic induction, 5 × 10^4^ cells were seeded and maintained in a chondrogenic induction medium (0.1 µM dexamethasone, 50 µg/mL L-ascorbic-2-2phosphate, 4 mg/mL L-proline, 1% insulin-transferrin-selenium (ITS), 10 ng/ml of the transforming growth factor (TGF)-β3, and 15% FBS) for 21 days^17,21,103^. Chondrogenic differentiation was confirmed by Alcian Blue staining for detecting glycosaminoglycans formation along with the chondrogenic mRNA markers (*Sox9* and *Col2a1*) expression by RT-qPCR.

Regarding adipogenic induction, 3 × 10^4^ cells were seeded and maintained in an adipogenic induction medium containing 0.1 mg/mL insulin, 1 µM dexamethasone, 0.5 mM 3-isobutyl-1-methylxanthine (IBMX), and 0.1 mM indomethacin^17,21,103^. After 28 days of induction, cells were stained with 0.1% Oil Red O, and adipogenic mRNA markers (*Leptin* and *LPL*) were analyzed.

### IPC induction by microenvironmental manipulation

In this regard, the three-step induction protocol modified from previously published reports was used^21,37,64^. Briefly, the cells were trypsinized and resuspended in a series of three pancreatic induction media, namely, serum-free medium (SFM)-A, SFM-B, and SFM-C. Cells were consequently maintained in SFM-A for 3 days (72 h), SFM-B for 2 days (48 h), and SFM-C for 5 days (120 h). SFM-A was SFM-DMEM/F12 or SFM-DMEM (basal medium) supplemented with 1% bovine serum albumin (BSA, Cohn fraction V, fatty-acid-free) (Sigma-Aldrich, USA), 1X insulin-transferrin-selenium (ITS) (Invitrogen), 4 nM activin A (Sigma-Aldrich), 1 nM sodium butyrate (Sigma-Aldrich), and 50 µM beta-mercaptoethanol (Sigma-Aldrich). SFM-B was a basal medium supplemented with 1% BSA, 1X ITS, and 0.3 mM taurine (Sigma-Aldrich). SFM-C was a basal medium containing 1.5% BSA, 1X ITS, 3 mM taurine, 100 nM glucagon-like peptide (GLP)-1 (Sigma-Aldrich), 1 mM nicotinamide (Sigma-Aldrich), and 1X nonessential amino acids (NEAAs) (Sigma-Aldrich). The gamma-secretase inhibitor (DAPT) was used in some experiments at 25 µM^21,40^.

Regarding culture maintenance, three different techniques were employed: low attachment, hanging-drop, and hydrogel (Matrigel)-embedded culture techniques. For a two- dimensional (2D) low attachment culture, 60 mm nontreated culture dishes (Eppendorf, USA) were used. 10^6^ cells were collected and suspended onto each dish using three induction media mentioned above. For the 3D hanging-drop culture, a GravityPLUS 96-well plate hanging-drop culture system (PerkinElmer, USA) was used. Cells were suspended in the induction media and seeded into hanging-drop wells at a concentration of 2 × 10^4^ cells per 40 µL per well. Another protocol was a 3D hydrogel-embedded culture. Cell colonies obtained from the hanging-drop culture were collected and embedded in hydrogel (Matrigel Matrix: growth factor reduced type) (Corning). In this regard, 100–150 µL of hydrogel and an induction medium mixture (1:1) was used to forming a dome-like structure onto each well of a 24-well culture plate (Corning). Cell Recovery Solution was used for gel digestion.

### IPC induction by genetic manipulation

Overexpression of *PDX1* by the lentiviral vector was used for the genetic manipulating approach. Lentivirus carrying *PDX1* was produced from the packaging of *pWPT-PDX1* (Addgene plasmid #12,256; gift from Didier Trono; http://n2t.net/addgene:12256; RRID: Addgene_12256)^39^, *psPAX2* (Addgene plasmid #12,260; gift from Didier Trono; http://n2t.net/addgene:12260; RRID: Addgene_12260), and *pMD2.G* (Addgene plasmid #12,259; gift from Didier Trono; http://n2t.net/addgene:12259; RRID: Addgene_12259) in human embryonal kidney (HEK 293FT) cells. The supernatant containing lentiviral particles were collected at 48- and 72-h post-packaging and filtered through a 0.45 µm filter. Viral particles were harvested using a Plasmid Midiprep Plus Purification Kit (Gene Mark Bio, Taiwan) and then freshly concentrated using an Amicon Ultra Centrifugal Filter (Merck Millipore, USA).

For the transfection protocol, cells at concentration of 5 × 10^4^ cells/well were seeded onto 24-well culture plates for 24 h and then treated with a 4 µg/mL polybrene infection/transfection reagent (Merck Millipore) for 30 min. Multiplicity of infection (MOI) at 20, 30, or 50 was used for each 24-h-transfection course.

### Reverse transcription-quantitative polymerase chain reaction (RT-qPCR)

RT-qPCR was used for mRNA analysis. The total RNA was collected using a TRIzol-RNA isolation reagent (Thermo Fisher Scientific Corporation) and extracted by a DirectZol-RNA isolation kit (ZymoResearch, USA) according to the manufacture’s protocol. RNA was converted to complementary DNA (cDNA) using ImProm Reverse Transcription System (Promega, USA). The amplification of targeted genes was carried out by FastStart Essential DNA Green Master (Roche Diagnostics, Switzerland) using a CFX96 Real-Time PCR Detection System (Bio-Rad) with specific amplification primers. Glyceraldehyde 3-phosphate dehydrogenase, *Gapdh*, was used as the reference gene. The relative mRNA expression of target genes was normalized with reference genes and control groups. The primer sequences were listed in Supplementary Table S1.

### Functional analysis for IPCs

Glucose-stimulated C-peptide secretion (GSCS) was used for the functional analysis of IPCs. Two glucose concentrations were used, 5.5 and 22 mM. Krebs–Ringer bicarbonate HEPES (KRBH) at pH 7.4 was used as a physiological buffer solution according to previous reports^21,106,107^. The KRBH buffer solution contained 120 mM NaCl, 5 mM KCl, 2.5 mM CaCl_2_, 1.1 mM MgCl_2_, 25 mM NaHCO_3_, and 10 mM HEPES. IPCs were gently collected and maintained with the KRBH buffer solution at 37℃ for 60 min as basal C-peptide secretion (0 mM glucose) and then respectively incubated in 5.5 mM (99 mg/dL) and 22 mM (396 mg/dL) glucose (Sigma-Aldrich) for 60 min each. The buffer solution in each incubation period was collected for measuring C-peptide concentration using canine C-peptide enzyme-linked immunosorbent assay (ELISA) kit (Merk Millipore) according to the manufacturing protocol. Secreted C-peptide levels were then normalized with the total DNA (ng) and incubation time (minutes). The total DNA was measured using the DNeasy Blood and Tissue Kit (Qiagen, CA) and a Qubit fluorometer (Thermo Fisher Scientific).

### Immunocytochemistry staining

IPC colonies were obtained and fixed in cold methanol for 15 min at 4℃ and then permeabilized by 0.1% TritonX-100 (Sigma-Aldrich) for 1 min at room temperature (RT). After that, background staining was reduced by incubating with 10% donkey serum for 1 h at 4℃. The primary antibodies, rabbit anti-human insulin (Cell Signaling Technology, USA)^108^ and mouse anti-rat glucagon (Abcam, USA)^109^, were used for overnight staining. Cyanine (Cy) 3-conjugated donkey anti-rabbit IgG (Bio Legend) and FITC-conjugated goat anti-mouse IgG (Bio-Rad) were used as a secondary antibody with respect to each primary antibody. DAPI was used for nuclear counterstaining. The results were observed under fluorescent microscope ZEISS Apotome.2 (Carl Zeiss, Germany) incorporated with Axio Observer Z1 and ZEN pro software (ZEISS International, Germany).

### Statistical analysis

The results were illustrated as whisker and box plot (N = 4). Statistical analysis was determined using SPSS Statistics 22 software (IBM Corporation, USA). The Mann–Whitney *U* test was used to compare two independent samples, while the Kruskal–Wallis test and a pairwise comparison were used to compare three or more groups. The significant difference was considered when the *p* value < 0.05.

## Supplementary Information

Supplementary Information 1.

Supplementary Information 2.

## Data Availability

The datasets generated and/or analyzed during the current study are available from the corresponding author upon reasonable request.
