# Supplementary material for: Tailored generation of insulin producing cells from canine mesenchymal stem cells derived from bone marrow and adipose tissue
Source: Sci Rep. 2021 Jun 11;11:12409. doi: 10.1038/s41598-021-91774-3 (PMC8196068; doi:10.1038/s41598-021-91774-3)
Supplement: Supplementary file 1 — Supplementary Information 1. [file 41598_2021_91774_MOESM1_ESM.pdf]

Supplementary information

Tailored generation of insulin producing cells from canine mesenchymal stem cells derived from bone marrow and adipose tissue

Watchareewan Rodprasert[1,2,3,5], Sirirat Nantavisai[2,3], Koranis Pathanachai[2,3,5], Prasit Pavasant[4], Thanaphum Osathanon[4], Chenphop Sawangmake[2,3,5,*]

[1]Inter-Disciplinary Program of Pharmacology, Graduate School, Chulalongkorn University, Bangkok, THAILAND

[2]Veterinary Pharmacology and Stem Cell Research Laboratory, Veterinary Stem Cell and Bioengineering Innovation Center (VSCBIC), Faculty of Veterinary Science, Chulalongkorn University, Bangkok, THAILAND

[3]Veterinary Stem Cell and Bioengineering Research Unit, Faculty of Veterinary Science, Chulalongkorn University, Bangkok, THAILAND

[4]Department of Anatomy and Center of Excellence for Regenerative Dentistry, Faculty of Dentistry, Chulalongkorn University, Bangkok, THAILAND

[5]Department of Pharmacology, Faculty of Veterinary Science, Chulalongkorn University, Bangkok, THAILAND

[*]e-mail: chenphop.s@chula.ac.th, chenphop@gmail.com

SUPPLEMENTARY TABLE

Supplementary Table S1 Primer sequences

Genes	Accession number	Sequences	5' 3'	Length (bp)	Tm (°C)
**Stemness genes**					
Zinc finger protein 42 (Zep42 or *Rex1)*	XM_003639567.1	Forward	AGGTTCTCACAGCAAGCTCA	199	59.24
		Reverse	CCAGCAAATTCTGCGCACTG		60.73
POU class 5 homeobox 1 (Pou5f1 or *Oct4)*	XM_538830.1	Forward	AGGAGAAGCTGGAGCAAAACC	100	60.55
		Reverse	GTGATCCTCTTCTGCTTCAGGA		59.50
**Proliferation marker**					
Proliferation marker protein Ki-67 (Ki67)	XM_014108788.1	Forward	GTGCAACTAAAGCACGGAGA	124	58.49
		Reverse	GAGATTCCTGTTTGCGTTTTCGT		58.49
**Osteogenic markers**					
Alkaline phosphatase (*Alp*)	NM_001197137.1	Forward	CCTGCCAGATAACTGCCTCT	168	59.16
		Reverse	GTGGAGACACCCATCCCATC		59.82
Runt-related transcription factor 2 (*Runx2*)	XM_005642335.1	Forward	GGAAGAGGCAAGAGTTTCACC	209	58.84
		Reverse	GTGCTCACTTGCCAACAGAA		58.89
Sp7 transcription factor (*Spp* or *Osx*)	XM_844688.3	Forward	GCGTCCTCCCTGCTTGAG	122	60.13
		Reverse	GCTTTGCCCAGTGTCGTTG		60.01
Secreted phosphoprotein 1 (*Spp1* or *Opn*)	XM_003434024.2	Forward	GCCACAGAGCAAGGAAAACTC	180	59.73
		Reverse	CTGCTTCTGAGATGGGTCAGG		60.13
Bone gamma-carboxyglutamate protein (*Bglap* or *Ocn*)[78]	XM_547536.4	Forward	GCCAGCCTATGGTCTCCTCTG	249	61.90
		Reverse	CCACCAGCTCCTTCTGTTCTCT		54.55
Collagen type I alpha 1 chain (*Col1a1*)	NM_001003090.1	Forward	CCAGCCGCAAAGAGTCTACAT	150	60.41
		Reverse	CTGTACGCAGGTGACTGGTG		60.67
**Chondrogenic markers**					

SRY (sex determining region Y)-box 9 (Sox9)	NM_001002978.1	Forward	TCTGGAGGCTGCTGAACGA	127	60.91
		Reverse	TTCTTCACCGACTTCCTCCG		59.40
Collagen type II alpha 1 chain (Col1a2)	NM_001006951.1	Forward	ATGAAAGACTGCCTCAGCCC	103	60.03
		Reverse	TCTGTCCCTTTGGTCCTGGT		60.40
**Adipogenic markers**					
Leptin (LEP)	NM_001003070.1	Forward	TGTGGCTTTGGCCCTATCTG	147	60.16
		Reverse	CAGCGACCCTCTGTTTGGAG		61.35
Lipoprotein lipase (LPL)	XM_005635734.3	Forward	CTGGAGAGACTCAGAAAAAGGT AAT	148	60.03
		Reverse	TCCTTCTGTAGATTTGCTCAGGT		60.40
**Pancreatic markers**					
<u>Pancreatic endoderm marker</u>					
Pancreatic and duodenal homeobox 1 (*Pdx1*)	NM_001284471.2	Forward	AAGTCTACCAAGGCTCACGC	201	60.04
		Reverse	GTGCCTCTCGGTCAAGTTCA		59.97
<u>Pancreatic beta-cell or insulin-producing cells (IPCs) markers</u>					
NK6 homeobox 1 (*Nkx-6.1*)	XM_544960.5	Forward	CAGGAGTTATGCAGAGCCCG	111	60.53
		Reverse	ACGTGGGTCTCGTGTGTTTT		60.11
ISL LIM homeobox 1 (*Isl-1*)	XM_848628.4, XR_001315955.1	Forward	TGGCTTACAGGCAAACCCAG	171	60.54
		Reverse	GACATCGACGCCACTTCACT		60.39
V-maf avian musculoaponeurotic fibrosarcoma oncogene homolog A (*Maf-A*)	XM_003431814.3	Forward	GCTTCAGCAAGGAGGAGGTC	136	60.39
		Reverse	CTCTGGAGCTGGCACTTCTC		60.11
Solute carrier family 2 (facilitated glucose transporter), member 2 (*Slc2a2* or *Glut-2*)	XM_545289.5	Forward	ACTCATCACAGGACGTGGAG	108	59.11
		Reverse	AGCTGAGTGTAGCGGTGAAG		59.76
Insulin (*Ins* or *Insulin*)	NM_001130093.1	Forward	TGGTAGAGGCTCTGTACCTGG	235	60.34
		Reverse	CGCCCCTAGTTGCAGTAATTC		59.06
<u>Pancreatic-relating markers</u>					

Glucagon (*Gcg* or *Glucagon*)	NM_001003044.1	Forward<br>Reverse	TCCAATCGCGGTGTCAGAAG<br>ACCCTGAGAATGACGCTTGT	197	60.39<br>59.31
Glucagon-like peptide 1 receptor (*Glp1r*)	XM_014118246.1	Forward<br>Reverse	CACGGTGGGCTATACACTCTC<br>AGGACGCAAACAGGTTCAGG	116	59.93<br>60.54
**Notch targeted genes**					
Hes Family BHLH Transcription Factor 1 (*Hes-1*)	XM_025478075.1	Forward<br>Reverse	GAGAAGGCGGACATTCTGGA<br>ACCTCGTTCATACACTCGCTG	137	59.46<br>60.14
Hes Related Family BHLH Transcription Factor with YRPW Motif 1 (*Hey-1*)	NM_001002953.1	Forward<br>Reverse	ACCTGAAAATGCTGCACACG<br>GCTGGGAGGCGTAGTTGTTA	195	59.69<br>59.75
**Reference gene**					
Glyceraldehyde 3-phosphate dehydrogenase (Gapdh)	NM_001003142.1	Forward<br>Reverse	CCAACTGCTTGGCTCCTCTA<br>GTCTTCTGGGTGGCAGTGAT	100	59.38<br>59.67

SUPPLEMENTARY FIGURE

Supplementary Figure S1 Functional property of cAD-MSC-derived IPCs with Notch signaling manipulation upon culture medium maintenance. Glucose-stimulated C-peptide secretion (GSCS) analysis of the cAD-MSC-derived IPCs with Notch signaling manipulation upon culture medium maintenance (0, 5.56, and 25 mM glucose) was illustrated. Bars indicate a significant difference (*, p-value < 0.05).
