# Supplementary figures and images for: Tailored generation of insulin producing cells from canine mesenchymal stem cells derived from bone marrow and adipose tissue
Source: Sci Rep. 2021 Jun 11;11:12409. doi: 10.1038/s41598-021-91774-3 (PMC8196068; doi:10.1038/s41598-021-91774-3)

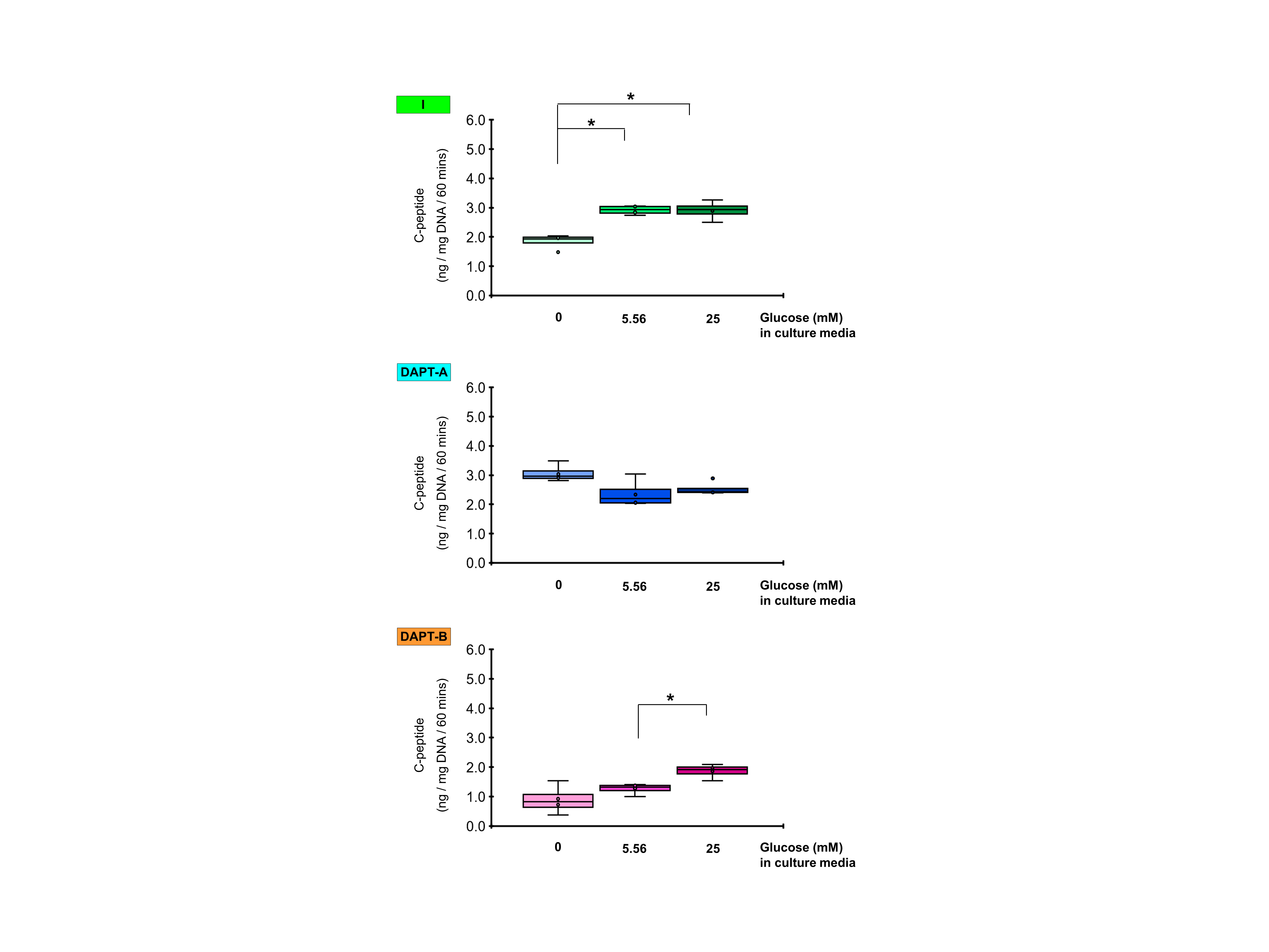

Supplement: Supplementary file 2 — Supplementary Information 2. [file 41598_2021_91774_MOESM2_ESM.tif]
